# Supplementary material for: Social determinants of health on all-cause and cause-specific mortality in US adults with chronic obstructive pulmonary disease: NHANES 2005–2018
Source: PLoS One. 2025 May 15;20(5):e0322654. doi: 10.1371/journal.pone.0322654 (PMC12080834; doi:10.1371/journal.pone.0322654)
Supplement: S1 Table — (DOCX) [file pone.0322654.s001.docx]

**S1 Table.** Definitions of social determinants of health domains and sub-items.

| **Domain** | **Sub-items** | **Categories** | **Definitions** |
| --- | --- | --- | --- |
| **Economic stability** | **Employment** | 0: Employed, student, or retired  1: Unemployed | Participants were asked what type of work they have done in the last week and, if not working, the main reason why. All participants not working were classified as unemployed, except those who responded they were a student or retired were grouped with those reporting employment. |
|  | **Family income-to-poverty ratio** | 0: ≥300%  1: <300% | Participants were asked their family income and size, and the poverty/income ratio is the ratio of family income to poverty. The Department of Health and Human Services poverty guidelines were used as the poverty measure to calculate this ratio. These guidelines are issued each year, in the Federal Register, for determining financial eligibility for certain federal programs. The guidelines vary by family size and geographic location. |
|  | **Food security** | 0: Full security  1: Marginal, low, or very low security | Participants responded to the U.S. Food Security Survey Module questions whether: 1) they were worried if food would run out before there was money to buy more; 2) the food they bought didn’t last and they didn’t have money to get more; 3) they couldn’t afford to eat balanced meals; 4) they had cut the size of meals or skipped meals because there wasn’t enough money for food; 5) if yes to #4, how often meals were cut or skipped; 6) they ate less than they felt they should because there was not enough money to buy food; 7) they were hungry but didn’t eat because they couldn’t afford food; 8) they lost weight because they didn’t have enough money for food; 9) they did not eat for a whole day because there was not enough money for food; 10) if yes to #9, how often they did not eat for the whole day. Levels of food security were classified as follows: full food security, no affirmative responses; marginal food security, 1-2 affirmative responses; low food security, 3-5 affirmative responses; and very low food security, 6-10 affirmative responses. |
| **Education access and quality** | **Education level** | 0: High school graduate or higher  1: Less than high school | Participants were asked for the highest grade or level of school they completed. The response categories are: less than 9th grade education, 9-11th grade education (includes 12th grade and no diploma), High school graduate/GED, some college or associates (AA) degree, and college graduate or higher. |
| **Healthcare access and quality** | **Access to healthcare** | 0: Yes  1: No | Participants were asked if there is a place they usually go when sick or needing advice about health. If they answered “yes” or “there is more than one place” to this question, they were classified as having a routine place for healthcare. If yes, but the facility is a hospital emergency room, they were classified as not having a routine place for healthcare. |
|  | **Health insurance** | 0: Private  1: Government or none | Participants were asked whether they are covered by health insurance or some other kind of health care plan. They are subsequently asked if covered by private insurance or several types of government insurance (Medicare, Medi-Gap, Medicaid, SCHIP, military health care, Indian Health Service, state-sponsored health plan, or other government insurance). |
| **Neighborhood and built environment** | **Housing instability** | 0: Own home  1: Rent home or other arrangement | Participants were asked if the home they are living in is owned, being bought, rented, or occupied by some other arrangement. A person was considered to own the home even if they are still paying on a mortgage. |
| **Social and community context** | **Marital status** | 0: Married or living with a partner  1: Not married or living with a partner | Participants were asked whether they were married, widowed, divorced, separated, never married, or living with a partner. Those reporting marriage or living with a partner were grouped together. |
