# Supplementary material for: Social determinants of health on all-cause and cause-specific mortality in US adults with chronic obstructive pulmonary disease: NHANES 2005–2018
Source: PLoS One. 2025 May 15;20(5):e0322654. doi: 10.1371/journal.pone.0322654 (PMC12080834; doi:10.1371/journal.pone.0322654)
Supplement: S2 Table — (DOCX) [file pone.0322654.s002.docx]

**S2 Table.** Cox regression analysis of SDoH (by tertile) and long-term mortality in patients with COPD: Sensitivity analyses.

|  | Crude model | | Model 1 | | Model 2 | |
| --- | --- | --- | --- | --- | --- | --- |
|  | HR (95%CI) | P | HR (95%CI) | P | HR (95%CI) | P |
| **All-cause mortality** |  |  |  |  |  |  |
| SDoH (multi-category) |  |  |  |  |  |  |
| Q1 | ref |  | ref |  | ref |  |
| Q2 | 2.302(1.807,2.93) | <0.0001 | 2.402(1.935,2.98) | <0.0001 | 2.013(1.617,2.50) | <0.0001 |
| Q3 | 1.700(1.315,2.19) | <0.0001 | 2.847(2.123,3.81) | <0.0001 | 2.105(1.575,2.81) | <0.0001 |
| P for trend |  | <0.0001 |  | <0.0001 |  | <0.0001 |
| **Cancer mortality** |  |  |  |  |  |  |
| SDoH (multi-category) |  |  |  |  |  |  |
| Q1 | ref |  | ref |  | ref |  |
| Q2 | 2.660(1.582,4.47) | <0.001 | 2.742(1.605,4.68) | <0.001 | 2.306(1.332,3.99) | 0.003 |
| Q3 | 1.799(1.057,3.06) | 0.031 | 3.085(1.607,5.92) | <0.001 | 2.252(1.205,4.21) | 0.011 |
| P for trend |  | <0.001 |  | <0.0001 |  | 0.001 |
| **Cardio-cerebrovascular disease mortality** |  |  |  |  |  |  |
| SDoH (multi-category) |  |  |  |  |  |  |
| Q1 | ref |  | ref |  | ref |  |
| Q2 | 1.686(1.032,2.75) | 0.037 | 1.821(1.155,2.87) | 0.010 | 1.406(0.749,2.63) | 0.289 |
| Q3 | 1.447(0.798,2.62) | 0.224 | 2.834(1.586,5.06) | <0.001 | 1.836(1.042,3.23) | 0.036 |
| P for trend |  | 0.094 |  | <0.001 |  | 0.040 |

Crude model: No adjustment for any potential influence factors.

Model 1: Adjusted for age, sex, ethnicity.

Model 2: Adjusted for age, sex, ethnicity, drinking status, smoke, BMI, DM, hypertension, CVD, cancer.

Abbreviations: CI, confidence interval; HR, hazard ratio. Abbreviations: SDoH, social determinants of health; COPD, Chronic Obstructive Pulmonary Disease; BMI, body mass index; DM, diabetes mellitus; CVD, cardiovascular disease.
